# 

**Published:** 1883-02

**Authors:** 


### CONTENTS.

***MISCELLANEOUS.*** PAGE

The I. H. A. Editorial, . . . . . . . . . . . . . 41
Reprints, . . . . . . . . . . . . . . . . . 43
Cinchona Officinalis; Provings. B. Fincke, M. D., . . . . . 44
Notes on Genito-Urinary Therapeutics, . . . . . . . . . 52
Merc. corr. in Dysentery, etc. Carroll Dunham, M. D., . . . . 53
Infallible Diagnosis! . . . . . . . . . . . . . 55
Dr. Holmes on Physical Diagnosis (extract), . . . . . . . 56
The *Medical Counselor* and the I. H. A. By an International, . . 57
Definitions. E. W. Berridge, M. D., . . . . . . . . . 61
Some Mental Symptoms Connected with the Catamenia, . . . . . 64

***CLINICAL BUREAU.***

Clinical Reflections. Ad. Lippe, M. D., . . . . . . . . . 68
Two Berberis Cases. E. Rushmore, M. D., . . . . . . . . 70

***NOTES AND NOTICES.***

REMOVALS. New York State Hom. Society—New Journal—The U. S. Medical Investigator—The Love Parasite—Errata, . . . . 71

***APPENDIX.***

Dysentery, . . . . . . . . . . . . . . . . 9
Cough Symptoms, . . . . . . . . . . . . . . 51--56

